# Font Matters: Deciphering the Impact of Font Types on Attention and Working Memory

**DOI:** 10.7759/cureus.59845

**Published:** 2024-05-07

**Authors:** Mahesh Arjundan Gadhvi, Anshika Baranwal, Ashwathreddy Chalakapure, Abhinav Dixit

**Affiliations:** 1 Physiology, All India Institute of Medical Sciences, Jodhpur, Jodhpur, IND

**Keywords:** cognition, sans serif, serif, working memory, n-back test, letter cancellation task, font type, attention

## Abstract

Introduction

Various types of fonts such as serif, sans serif, and script are used during writing and reading, which can affect the reader's attention and working memory, though there is only a subtle difference at the end of the letter. The study aimed to see the effect of font type on working memory and attention.

Methods

The study included healthy male adults between 18 and 40 years of age. After taking all the necessary precautions, a letter cancellation test and 2-back task in serif, sans serif, and script font types were done to evaluate subjects' attention and working memory.

Results

A total of 30 subjects participated in the study. The letter cancellation task (LCT) was statistically significant (P<0.05) between the three groups, where the time taken to complete the task was the shortest for serif fonts, indicating heightened attention to serif fonts. However, the accuracy of the N-back test did not show statistically significant differences (P>0.05) among the three font groups, indicating no significant change in working memory.

Conclusion

The type of font used can impact the reader's attention, with Times New Roman font demonstrating greater attention, particularly in the context of the letter cancellation task.

## Introduction

Nowadays, pen-paper-based writing and reading are replaced by digital material, and daily communication happens with the help of messages on mobile phones or computers [1]. There are both computer-based and paper-pencil-based tasks for cognitive assessment. Paper-pencil-based tasks have the advantage that they can be used anywhere and are easy to administer and relatively inexpensive. As good handwriting can impact the reader's attention, the type of font used in the digital script can also affect reading. A literate person goes through reading, writing, and understanding in different fonts during day-to-day activities. The growing variety of languages changes the perception of reading, including the speed of reading and understanding, as well as attention [2,3]. The use of the English language has increased [4], and we come across a wide variety of fonts in day-to-day activities such as reading books, newspapers, and mobile phones where numerous different fonts are used.

Carefully selected font type for instructions and training is important for safety and survival, as is the case with disaster-related instruction. For education-related activities such as memory retention after reading textbooks or making lecture notes, font type can make a difference. For professional job applications, font type can make a massive difference if wisely chosen font type is used, affecting readers' attention [5]. Broadly, fonts can be divided into serif, sans serif, and script according to the stroke style. While serif and sans serif fonts seem to have only a slight difference, a simple line at the end of a stroke significantly affects reading and comfort [6].

The retention of any information can be done in the form of long-term memory, short-term memory, or working memory. Some information can be memorized for seconds only, which can come under working memory [7]. Working memory can have three components: the phonological loop, visuospatial sketch pad, and central executive system. Speech or acoustic information storage for the temporary period is a phonological loop, while visual information storage comes under visuospatial memory, and the information processing and attention come under the central execution function [8].

Working memory can be assessed by various computer and pen-paper-based tasks such as complex span tasks, updating tasks, recall N-back tasks, binding tasks, secondary memory tasks, or reasoning tasks [9], out of which the N-back test is the specific computer-based task routinely used [10]. In the N-back task, different alphabets are shown on the screen, and the task's font type can change the working memory of the individual. A person's attention toward tasks and ignoring irrelevant information is critical in cognitive performance [11]. During reading or writing, attention should be maximum to improve productivity. As discussed earlier, a variety of font types can affect the reader's attention. Arithmetic tests, trail-making tasks, forward or backward digit span, digit symbol coding, letter cancellation task (LCT), and spatial span are some tasks to measure attention in clinical research [12]. The letter cancellation task is a pen-paper-based task, which assesses attention that can be affected by font type.

We wish to compare serif, sans serif, and script fonts by the N-back test and letter cancellation task (computer- and pen-paper-based tasks) to see their effect on memory and attention. The effect of font type on working memory and attention is assessed by N-back and letter cancellation tasks.

## Materials and methods

The study aimed to see the effect of font type on working memory and attention. The Institutional Ethics Committee of All India Institute of Medical Sciences, Jodhpur, approved the study (AIIMS/IEC/2023/4302). It employed a cross-sectional design and was conducted at the cognitive neurophysiology laboratory of our institute. Healthy male subjects aged 18-40 years with a minimum of five years of schooling were recruited for the study after obtaining informed consent. The language proficiency score was measured using a Language Experience and Proficiency (LEAP) Questionnaire (LEAP-Q), and those volunteers with a score of 5 and above were recruited (Appendices).

The participants were asked to abstain from the consumption of stimulants such as caffeine for four hours before the test administration. Participants with a history of any neurological disease/head trauma that can affect attention or memory, subjects with color blindness, and participants taking medicines causing sedation/alcohol were excluded from the study because they can alter the attention and working memory of the subject.

Cognitive tasks

N-Back Test

For the computerized N-back test, the participants were made to sit comfortably in a chair with the monitor being placed at a distance of 25 cm. The N-back test was programmed using the SuperLab 4.0 software (Cedrus Corporation, San Pedro, California). A 2-back task was used for the assessment of working memory. English alphabets using different fonts (serif, sans serif, and script fonts) on small cases were displayed for 1500 ms duration, followed by 500 ms cue (+). Twenty-five percent of the alphabets in a block were hit. The participants were asked to press the spacebar when they found the hit and should skip the clicking of the spacebar when there was no hit. The accuracy of the N-back task was assessed using the total number of correct hits and correct skips out of the total alphabet in percentage.

Letter Cancellation Task

In the letter cancellation task, the participants have to search for and mark the target letter, in our study alphabet "a," from many different letters from left to right and from top to bottom. The task must be done as fast as possible with maximum accuracy. The task was given on A4 paper with a font size of 14. After completing the N-back task, the participants rested for five minutes and started the letter cancellation task. Twenty percent of the alphabets were hits. Overall, the time taken to complete the task was noted in seconds.

After completing the task in one font type, the participants had to wait for five minutes and after that two tasks were carried out in other font types. Randomization was done for different font types. Times New Roman font (sans serif), Calibri Body (serif), and Lucida Script fonts (script) were employed for the study.

Statistical analysis

The data was analyzed using Prism 10™ (GraphPad Software, San Diego, CA). One-way repeated measures analysis of variance (ANOVA) test was employed for statistical analysis. A comparison of all three font types was done with mean percentage accuracy for the 2-back test and mean time to complete the letter cancellation task. The average number of omissions in the letter cancellation task is also compared. If the p*-*value is less than 0.05, the result is considered statistically significant.

## Results

The present study was conducted on 30 healthy males with a mean age of 31±2.3 years. In the 2-back task, mean percentage accuracy was analyzed for different fonts, and the results are depicted in Table 1.

**Table 1:** Mean percentage accuracy of serif, sans serif, and script fonts for the 2-back task

Type of font	Mean percentage accuracy (mean±standard deviation)	P-value
Serif	93.67±6.33	0.2136
Sans serif	92.83±7.09	
Script	91.91±5.40	

Data analysis revealed a nonsignificant difference (P>0.05) in the mean percentage accuracy for different fonts. The mean time to complete the letter cancellation task was analyzed in Table 2.

**Table 2:** Mean time to complete the letter cancellation task in serif, sans serif, and script fonts

Type of font	Mean time to complete the letter cancellation task in seconds (mean±standard deviation)	P-value
Serif	161.5±26.76	0.0129
Sans serif	170.9±28.30	
Script	171.2±30.60	

There was a significant difference in the mean time to complete the test, with the shortest time being in serif font and the longest in script font.

The number of omissions was also analyzed in LCT (Table 3), where the same trend was repeated with serif font with the least omissions and sans serif with the highest omissions.

**Table 3:** Average number of omissions during the letter cancellation task in serif, sans serif, and script fonts

Type of font	Average number of omissions in the letter cancellation task
Serif	2.36
Sans serif	3.48
Script	2.92

## Discussion

The present study evaluated the effect of fonts on the 2-back task and on the letter cancellation task. Our study found no effect of font on 2-back tasks. However, there was a significant difference in the Mean time to complete the letter cancellation task.

Font type had a significant effect on attention in the form of time to complete LCT in our study, and serif fonts had the least time to complete LCT with the least number of omissions. In contrast, script fonts posed a substantially greater challenge to the participants. This extended duration to complete the task hinted at the additional cognitive load imposed by script fonts, which can be due to their elaborate design.

The effect of font type on working memory and attention is studied using various techniques. Haque et al. [13] compared serif, sans serif, script, and monograph fonts on comprehension and memory, where serif was the easiest font type to comprehend. In contrast to the above results, Akhmadeeva et al. found no difference in comprehension between serif and sans serif font types when used in Cyrillic script [14]. In our study, we found that serif fonts did not affect the memory of the individual.

Reading charts in ophthalmology printed in either Helvetica Neue (T1) Roman sans serif (Adobe) or Times New Roman PS Roman serif (Adobe) was used to investigate the effect of font type by Radner et al. where there was no statistical significance in reading acuity, reading speed, and other reading parameters [15].

The mean percentage accuracy of the N-back task was different for various font types in our study, but the lack of statistical significance denotes no alteration in working memory during the use of different font types. To investigate the effect of font type on memory, Upchurch displayed three sets of instructions in serif, sans serif, and script font type and asked the participants to place the instructions back in the order in which they were originally presented. They concluded that a serif font is superior to a sans serif or script font [16]. However, in our study, we did not find any significant difference in working memory between the three font types.

Communication through text is growing, including in scientific journals where abstracts are used to communicate. Kaspar et al. studied the effect of serif and sans serif fonts on comprehensibility, interest in reading, and overall appeal of abstracts in 188 students, where they found that abstracts written in serif fonts had increased the participants' interest [17]. We also found increased attention in serif fonts during LCT.

Banerjee and Bhattacharyya studied the effect of serif and sans serif font types with 10, 12, and 14 font sizes in two groups. They recorded reading time, ranking, and overall mental workload during reading 18 paragraphs where they concluded that Courier New (serif) with a font size of 14 was associated with increased reading speed [5]. These results were the same as our study where in LCT, the time to complete the task was the lowest for the serif font type.

The effect of font type on readability is also well-documented in a study by Tarita-Nistor et al., where MNRead font charts (Lighthouse Low Vision Products, Long Island City, NY) in different font types were used for the assessment of reading performance in patients with age-related macular degeneration. They found that the Courier font type increases reading speed significantly compared to the Times New Roman font type [18]. But the speed was lowest for the Arial font type, which is sans serif. Overall, serif fonts had greater performance than sans serif fonts, which was in line with our findings.

Though many studies indicated higher attention with serif fonts, there are some studies that state no difference in attention with different fonts. Perea did not find any difference in reading time and eye tracking during paragraph reading in serif and sans serif fonts [19]. These studies used different methodologies to measure attention or readability.

The results of the study were significant, but the study has some limitations. Only one test for the assessment of each domain was used in this study, which can be assessed by more such tasks in the subsequent studies. Also, the results are specific to one region only. Multicentric studies can be carried out in the future to justify the results.

## Conclusions

As there is the availability of various types of fonts to write and read, the type of font used can impact the reader's attention. Times New Roman, a serif type of font, demonstrated greater attention, particularly in the context of the letter cancellation task, whereas font types do not affect working memory assessed by the N-back task; thus, we can conclude that serif fonts can be used by the writer to grab readers' attention.

## Figures and Tables

**Figure 1:**
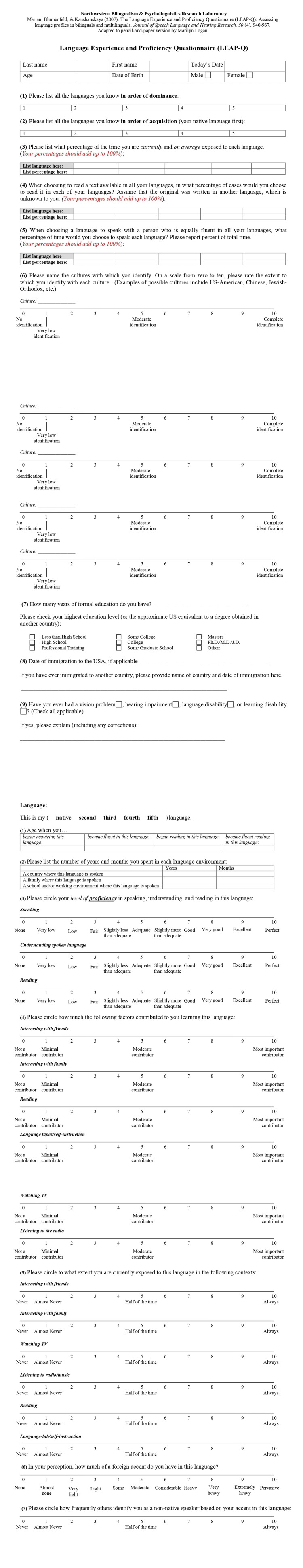
LEAP Questionnaire used for the assessment of language proficiency LEAP: Language Experience and Proficiency

## Appendices

Figure 1 shows the LEAP Questionnaire used.
